# A Review of Cardiovascular Toxicity of Microcystins

**DOI:** 10.3390/toxins11090507

**Published:** 2019-08-30

**Authors:** Linghui Cao, Isaac Yaw Massey, Hai Feng, Fei Yang

**Affiliations:** Department of Occupational and Environmental Health, Xiangya School of Public Health, Central South University, Changsha 410078, Hunan, China

**Keywords:** microcystins (MCs), toxicity, cardiovascular disease (CVD)

## Abstract

The mortality rate of cardiovascular diseases (CVD) in China is on the rise. The increasing burden of CVD in China has become a major public health problem. Cyanobacterial blooms have been recently considered a global environmental concern. Microcystins (MCs) are the secondary products of cyanobacteria metabolism and the most harmful cyanotoxin found in water bodies. Recent studies provide strong evidence of positive associations between MC exposure and cardiotoxicity, representing a threat to human cardiovascular health. This review focuses on the effects of MCs on the cardiovascular system and provides some evidence that CVD could be induced by MCs. We summarized the current knowledge of the cardiovascular toxicity of MCs, with regard to direct cardiovascular toxicity and indirect cardiovascular toxicity. Toxicity of MCs is mainly governed by the increasing level of reactive oxygen species (ROS), oxidative stress in mitochondria and endoplasmic reticulum, the inhibition activities of serine/threonine protein phosphatase 1 (PP1) and 2A (PP2A) and the destruction of cytoskeletons, which finally induce the occurrence of CVD. To protect human health from the threat of MCs, this paper also puts forward some directions for further research.

## 1. Introduction

The cardiovascular system is considered a dynamic system. As the first organ in embryonic development, the heart provides nutrients and oxygen to all of the various organs and tissues. The normal functions of the cardiovascular system are mainly affected by genetic factors, environmental factors and the interaction between the two factors, which is considered a principal cause of cardiovascular disease (CVD) [1]. It is estimated that about 290 million patients suffer from CVD [2]. Additionally, the recent epidemiological survey indicated that the prevalence and mortality of CVD in China is still on the rise, and the mortality rate of CVD is the highest, in comparison to tumors and other diseases, accounting for more than 40% of the total number of deaths resulting from diseases in China [2]. The burden of CVD is increasing, which has become a major public health problem. Thus, it is important to take measures towards preventing and curing of CVD.

The increasing eutrophication and frequent cyanobacterial blooms in water bodies have become a severe public health concern globally. Cyanobacterial blooms are usually characterized by the mass formation of cyanobacteria, green algae and diatom, leading to the death of aquatic organisms due to hypoxia. In addition, some algae (blue-green algae) including *Microcystis*, *Anabaena*, *Anabaenopsis*, *Oscillatoria* and *Nostoc* [3,4,5,6] can produce various potent toxins, such as cyclic peptides, alkaloids and lipopolysaccharides, in the process of metabolism [7]. Subsequently, negative human and animal health effects may be induced when exposed to these toxins [8,9]. Among the cyanotoxins produced during cyanobacterial blooms, monocyclic peptide microcystins (MCs) are the most abundant, widely distributed, extremely toxic and difficult to remove when dissolved in water bodies [10,11]. 

**Figure 1:**
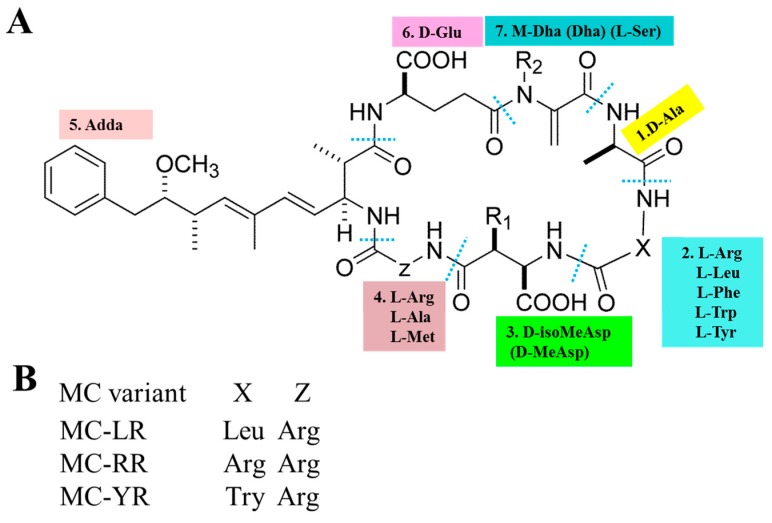
General chemical structure of MCs (microcystins). (**A**) 1–7 represent seven amino acid residues, respectively. *X* and *Z* in positions two and four are highly variable L-amino acids that determine the suffix in the nomenclature of MCs. (**B**) represents some of the most frequent MC congeners (adapted from Chen et al. [19]).

The basic chemical structure of MCs is cyclo-(D-Ala 1-X 2-D-MeAsp 3-Z 4-Adda 5-D-Glu 6-Mdha 7) (as shown in Figure 1A). In addition, (2S, 3S, 8S, 9S)-3-Amino-9-methoxy-2,6,8-trimethyl-10-phenyldec-4,6-dienoic acid (Adda) is critical for the toxicity of MCs, since this region is found only in cyanobacterial peptides (MCs and nodularins) and is vital for the interactions with protein phosphatases. X and Z in positions 2 and 4 are variable L amino acids. D-MeAsp 3 is D-erythro-β-methylaspartic acid and Mdha is N-methyldehydroalanine [12]. The alterations in amino acids have resulted in the identification of over 200 MC isomers [13]. The main differences lie in the two L-amino acids at positions 2 and 4 as well as the methylation or demethylation of D-Me Asp and M-Dha. The most common congeners are MC-LR (Microcystin-LR), MC-RR (Microcystin-RR) and MC-YR (Microcystin-YR), with different combinations of Leucine (L), arginine (R) or tyrosine (Y) (as shown in Figure 1B). MC-LR is the most toxic isomer with a contribution of 46–99.8 % of the total MCs in natural waters [14,15,16]. Although MC-RR and MC-YR are also widely distributed, their toxicities are second to MC-LR [17,18].

Studies have shown that human beings are exposed to MCs mainly through drinking water, body contact, long-term use of seafood and algal dietary supplements, where drinking water is the principal route [8]. To minimize the health hazard caused by MCs, the World Health Organization (WHO) specifies that the maximum allowable content of MCs in drinking water should not exceed 1 μg/L [20]. Research indicated that MCs negatively affect various human organs when exposed to it. Several organs or tissues including liver [21,22,23,24,25,26], kidney [25,26,27], nervous system [28,29,30], gastrointestinal tract [31,32,33], reproductive system [34,35] and cardiovascular [36] have been reported to be target organs for MC toxicity. This review will focus on the cardiovascular toxicity of MCs and provide some evidence of cardiovascular toxicity caused by MCs. To protect human health from the threat of MCs, this paper also puts forward some directions for further research.

## 2. Cardiovascular Toxicity of MCs

**Figure 2:**
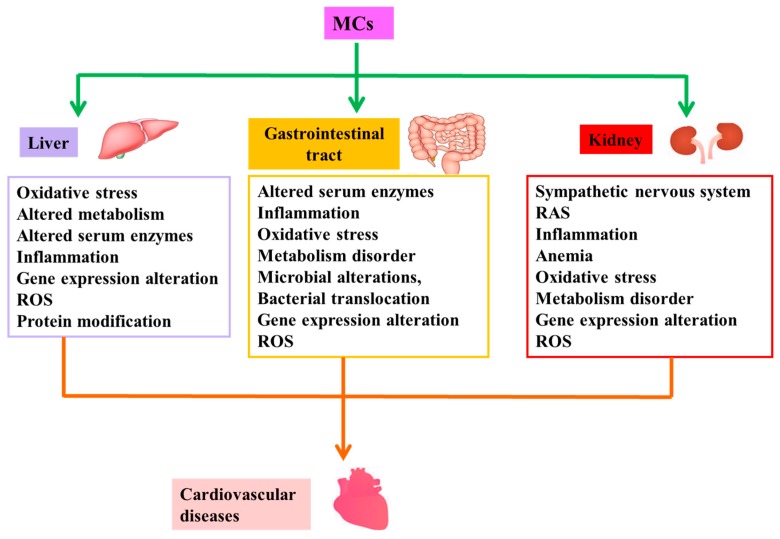
Possible mechanisms of indirect cardiovascular toxicity induced by MCs. MCs are capable of causing cardiovascular disease by inducing pathological changes in structure and/or function of liver, gastrointestinal tract and kidney, namely indirect cardiovascular toxicity of MCs. Abbreviation: RAS: renin-angiotensin system; ROS: reactive oxygen species.

To date, a large number of studies have shown that MCs can induce cardiovascular toxicity in vivo and vitro directly [36,37,38,39,40,41,42,43,44,45,46,47,48,49,50,51,52,53]. Table 1 and Table 2 represent summaries of cardiovascular toxicity in in vivo and vitro studies, respectively. That is, MCs have the potential to cause CVD directly. Numerous investigators have also demonstrated that MCs are capable of causing CVD by inducing pathological changes in structure and/or function of other target organs or tissues [21,22,23,24,25,26,27,31,32,33,54,55,56,57,58,59,60,61,62], namely indirect cardiovascular toxicity of MCs. Figure 2 illustrates the possible mechanisms of indirect cardiovascular toxicity induced by MCs.

Abbreviations in Table 1 and Table 2: SDH: succinate dehydrogenase; MMP: mitochondrial membrane potential; LDH: lactic dehydrogenase; AST: aspartate aminotransferase; CK: creatine kinase; GSH: glutathione; GPx: glutathione peroxidase; ALP: alkaline phosphatase; ALT: alanine aminotransferase; RBC: red blood cell counts; MCH: mean corpuscular hemoglobin; MCV: mean corpusular volume; MCHC: mean corpusular hemoglobin concerntration; HGB: hemoglobin; HCT: red blood cell specific volume; WBC: white blood cell counts; Mon: monocyte; Gran: granulocyte; PCNA: proliferating cell nuclear antigen; ROS: reactive oxygen species; SOD: superoxide dismutase; MDA: malondialdehyd; NRF 2: erythroid-like 2; HO 1: heme oxygenase 1; CAT: catalase; GST: glutathione s-transferase; GLT: glutamate transporter; AchE: acetyl cholinesterase.

### 2.1. Direct Cardiovascular Toxicity

This implies that MCs can affect the cardiovascular system, including all tissues, cells, blood and vascular in the heart, directly, and result in abnormal structure and/or functioning of the cardiovascular system.

#### 2.1.1. Transportation of MCs

Researchers have demonstrated that MCs can be transported into the various cells through organic anion transporting polypeptides (OATP) after exposure [63]. Almost every organ is capable of expressing the OATP family genes [8,64], though some OATP genes are expressed preferentially in specific tissues, or even selectively expressed [65]. Heart tissue is no exception [66]. Several OATP family genes including OATP4A1, OATP2A1, OATP2B1 and OATP3A1 have been reported to be expressed in heart tissue [67,68]. This signifies that MCs can be transported to and accumulated in cardiac tissue cells, and finally jeopardizing them, though the mechanisms of MCs entering cardiomyocytes await further exploration [69]. Moreover, MCs have been confirmed to enter into the cell depending on the degree of blood perfusion and types and expression levels of OATP carriers [70].

#### 2.1.2. Cytoskeleton Disruption and Mitochondrial Dysfunction

The cardiomyocytes, fibroblasts, telocytes, mast cells, endothelial cells, white blood cells and other immunologically cells (including smooth muscle cells, adipocytes and pericytes) are documented as cells in the cardiac tissue [71]. Among the cells, cardiomyocytes are reported to be rich in mitochondria, accounting for about 40% of the volume of myocardial cells [71]. Kowaltowski et al. demonstrated that the large number of unsaturated fatty acids on the mitochondrial membrane made cardiomyocytes vulnerable to free radicals and prone to the occurrence of oxidative stress [72]. Oxidative stress is related to the pathophysiology of many cardiomyopathies, such as anthracycline mediated cardiomyopathy [73] and alcoholic cardiomyopathy [74]. Goffart et al. declared that mitochondrial defects contributed to cardiomyopathy and heart failure (HF) [75]. An increasing number of studies have also confirmed that MCs can cause oxidative stress imbalance in mitochondria, further resulting in infiltration of neutrophils in tissues, raising secretion of protease and producing substantial oxidative intermediates, thus conducing to cell aging and even death [76,77,78,79,80]. 

In 2001, for the first time, Zhang et al. [37] proved that MC-LR could induce cardiotoxicity. Short-term toxic effects of MC-LR on SD (Sprague-Dawley) rats by intraperitoneal (I.P.) injection with different doses of MC-LR was investigated, and the results indicated that MC-LR could damage the physical structure of cardiomyocytes and alter biochemical parameters, including lactic dehydrogenase, aspartate aminotransferase and creatine kinase (CK) [37]. Subsequently, a follow up study was conducted and MC-LR was confirmed to have an involvement in pericardial edema and tubular heart formation in loach, Misguruns mizolepis Gunthe embryos [38]. In a chronic study [39], Wistar rats were injected with 10 μg/kg MC-LR by I.P. every two days for eight months and MC-LR treated animals exhibited no remarkable change in appearance and morphology of the heart. Although the results of TUNEL (terminal deoxynucleotide transferase-mediated deoxy-UTP nick end labelling) assay demonstrated no alteration in apoptosis, the cytoskeletons of cardiomyocytes were destructed, which were characterized by a loss of cell crossstriations, lower myofibril volume fraction, enlargement of cardiomyocytes volume, decrease of myofibrillar volume and even fibrosis, and infiltration of mononuclear in the interstitial tissue [39]. In 2010, a similar chronic study was carried out using MC-YR by Suput et al. [40]. Results showed that the volume and density of myocardium decreased with fiberous proliferation, and a few of them were infiltrated by lymphocytes. In addition, larger cardiomyocytes and abnormal nuclear structure were found, but the TUNEL assay results revealed no increase in apoptosis. Taken together, these results suggested that long-term exposure to relatively low doses of MC-LR and MC-YR can induce myocardial atrophy and fibrosis. 

Acute exposure to MCs has also been demonstrated to induce cytoskeleton disruption and mitochondrial dysfunction. In an attempt to investigate the toxicity of MC-LR to the heart by I.P. injection into rats with concentrations 0.16 LD_50_ (14 g/kg) and 1 LD_50_ (87 g/kg), Qiu et al. [41] reported myocardial infarction in almost all dead rats, and the surviving rats displayed a decrease in heart rate and blood pressure, which tend out to be associated with myocardial mitochondrial dysfunction. Further microscopic examination of pathological ultrastructure showed loss of adhesion between cardiomyocytes and swelling or rupture of mitochondria, and biochemical index test exhibited raising levels of CK and troponin I, which suggested an existence of cardiomyocytes damage. In addition, the level of lipid peroxide was notably increased, prompting the occurrence of severe mitochondrial oxidative stress. Additionally, the respiratory chain enzyme complexes I and III were found to be inhibited, indicating that the mitochondrial electron transfer chain was blocked. Zhao et al. [36] injected MC extracts (mainly containing MC-LR and MC-RR) into the abdominal cavity of rabbits at the dose of 12.5 and 50 μg MC-LR eq/kg bw (body weight) and detected the ultrastructure and enzyme activity in mitochondria after hours of injection. Morphological changes of cardiac mitochondria, increased concentrations of lipid peroxide and activity of succinate dehydrogenase were observed. Nicotinamide adenine dinucleotide (NADH) dehydrogenase was also found to be inhibited, which further affected the mitochondrial electron transfer chain. Moreover, MCs could alter the activities of Ca^2+^-Mg^2+^-ATPase in mitochondria, thus destroying the ion homeostasis, which may conduce to the loss of mitochondrial membrane potential (MMP), and finally succeed in damnifying mitochondria of myocardial cells.

Wang et al. in a recent publication have provided a new interpretation of the possible role of MCs in the toxicological mechanisms of vascular dysplasia in vivo and in vitro [42]. Zebrafish juvenile and human umbilical vein endothelial cells (HUVECs) were co-cultured with MC-LR at the dose of 0.1 µM and 1 µM respectively. In vivo, MC-LR resulted in angiodysplasia, damaged vascular structures, reduced lumen size and blood flow as well as vascular dysfunction, while in vitro, apoptosis, activity of caspase3/9, mitochondrial ROS and p53 were increased, whereas MMP and proliferating cell nuclear antigen were inhibited. To explore the effect of MC-LR on cardiopulmonary system, Martins et al. [43] injected 100 μg MC-LR/kg into trahira, Hoplias malabaricus and reported that MC-LR induced cytotoxicity by enhancing oxidative stress and activity of related enzymes, and consequently affected cardiopulmonary function. This was similar to what Qiu et al. demonstrated [41]. In addition, MC-LR has recently been confirmed to inhibit the heart rate of Japanese Medaka (Oryzias Latipes) [44]. Currently, Xu et al. [49] provided an explanation of the possible role of antioxidant enzymes in the toxicological mechanisms of MCs at the transcription level by treating H_9_C_2_ (a kind of rat cardiomyocyte) cell lines with 10 μM MC-LR. The expression levels of cardiac rhythm and antioxidant genes were detected and the results suggested that the expression levels of rhythmic genes (*baml1, cry1, cry2, per1, per2*) were inhibited, while the antioxidant genes (*catalase, ho-1, sod1, sod2*) were upregulated. These results indicated that alteration in the rhythm of cardiomyocytes is one of the possible cardiac toxilogical mechanisms of MCs. 

The above evidence suggests that MCs can directly lead to cardiac malformation in larva, and induce myocardial atrophy and fibrosis in adults, although the mechanisms of the effect need to be further explored. MCs may also induce cardiovascular toxicity by transforming the morphology of cardiomyocytes, cell proliferation and apoptosis, cytoskeleton and cell rhythm, as well as the ultrastructure, oxidative stress, membrane potential and enzyme activity of respiratory chain in mitochondria of cardiomyocytes.

#### 2.1.3. Endoplasmic Reticulum Dysfunction

MCs are also known to give rise to CVD by damaging the endoplasmic reticulum (ER). ER is a tunneling system composed of membranes in eukaryotic cells, and it is an important organelle for protein synthesis, folding and secretion [81,82]. The stability of the ER environment is an essential precondition for realizing ER function. When the internal or external microenvironment of ER changes, the imbalance function of homeostasis may be caused, resulting in the occurrence of endoplasmic reticulum stress response (ERS) [83,84]. Ischemia-reperfusion injury, homocysteine and other chemicals’ treatment, protein synthesis abnormality, protein folding capacity dysregulation, ER calcium metabolism disorder, physicochemical or genetic factors, such as the disturbance of lecithin synthesis, are capable of arousing ERS [85,86]. Moderate stress was able to protect cells through unfolded protein response, while prolonged or excessive stress could trigger ER CHOP, JNK and Caspase-1/2, Ca^2+^ pathways to induce apoptosis [87]. This suggests that cardiomyocytes damage can be produced by interfering with ER stress-related pathways. Previous data indicated that ERS evoked from lipid overload, changes in redox state, free radicals and other physical and/or chemical factors, could cause apoptosis of endothelial cells and monocytes entering the vascular endothelium to engulf lipid and lipid foam cells formation, thus giving rise to CVD [88]. In summary, the occurrence and development of CVD such as atherosclerosis [89,90], diabetic heart disease [91], hypertension [92,93], myocardial hypertrophy and HF [94,95] are closely related to ERS.

In recent years, a considerable number of studies have demonstrated that MC-LR can induce ERS. Qi et al. [45] found deformed morphology, stunted growth, suppressed heart rate and increased apoptosis when zebrafish juveniles were treated with 4.0 mM MC-LR for 96 h. Further studies on the mechanism showed that the above phenotypes could be partially rescued by tauroursodeoxycholic acid (TUDCA, 20 mM, an inhibitor of ERS), which suggested that the developmental toxicity of MC-LR was possibly produced by activating ERS. That is, MC-LR could induce developmental toxicity and apoptosis by increasing ER oxidative stress. In addition, Cai et al. [96,97] demonstrated that the raise of ER stress and apoptosis had an involvement in neurotoxicity caused by MC-LR in rats. A study conducted by Zhao et al. [98] provided an explanation of the possible role of oxidative stress and ER stress in the toxicological mechanisms of MCs at the proteomic level, and the results revealed that MC-LR remarkably altered the abundance of 49 proteins that were involved in oxidative phosphorylation, cytoskeleton, metabolism, protein folding and degradation. Although there is no direct evidence that MC-LR can induce ERS in the cardiovascular system, it is reasonable to infer that MC-LR is capable of affecting cardiovascular function by activating ERS.

#### 2.1.4. Inhibition of PP1 and PP2A

Previous research exhibited that the main mechanism of MCs producing their toxic effects is to inhibit serine/threonine protein phosphatase 1 (PP1) and 2A (PP2A) by interacting with the subunits of serine/threonine protein phosphatase (PP), which result in the disruption of the dynamic equilibrium of protein phosphorylation as well as expression and activation of their downstream proteins, and further leading to the cytoskeletal reorganization [8,99,100,101,102,103,104]. PP regulates a series of processes in mammalian cells, including cell proliferation, division, signal transduction and gene expression [105]. In the cardiovascular system, reversible protein phosphorylation is central to a variety of cardiac processes such as excitation–contraction coupling, Ca^2+^ handling, cell metabolism, myofilament regulation and intercellular communication [100]. PP1 and PP2A have been reported to regulate the cardiac function by dephosphorylating a disparate collection of target proteins, Cav1.2 and ATP sensitive Na^+^/K^+^ channels [101,102,106,107,108,109]. Overexpression of PP2A-A subunit or its active substitute PP2B was found to induce myocardial hypertrophy or HF [110,111,112]. Similarly, Meyer-Roxlau et al. [103] in a recent investigation reported that the increasing activity of PP1 contributed to cardiac hypertrophy, HF and atrial fibrillation. Furthermore, PP might as well directly dephosphorylate some signal molecules or transcription factors to give rise to the occurrence of CVD [113,114]. Since a considerable number of data have indicated that MCs inhibited the activities of PP1 and PP2A [8,19,22,29], it is profitable to infer that the inhibition activities of PP1 and PP2A is one of the possible cardiac toxicological mechanisms of MCs. 

#### 2.1.5. Hemodynamic Alterations and Vascular Lesions

The heart is known to be rich in vessels and bloods, and vessels are the carrier of blood flow. The heart supplies blood to the body, providing power for the body’s normal metabolism [115,116]. Blood flow alterations and vascular lesions can jeopardize the function of the heart. LeClaire [46] noted a continuous decrease of heart rate, cardiac output (CO), stroke, oxygen consumption, carbon dioxide production, metabolic rate, accompanied progressive hypothermia and disrupted equilibrium of acid–base when male Fischer 344 rats were administered with I.P. injection of MC-LR at a dose of 100 μg/kg bw. Huang et al. [47] investigated the response indices to toxic MC-LR in the blood of mice with I.P. injection at different MC-LR concentrations and indicated that the phagocytic index, ROS, hematology of the majority of blood cells and volume of erythrocytes were influenced by the toxin. In addition, the alterations of some cytokines and ROS of leukocytes were observed [47]. Another in vivo study also showed a significant difference in red blood cell parameters (red blood cell counts, haematocrit values, mean corpuscular hemoglobin, mean corpusular volume and mean corpusular hemoglobin concerntration) when rats were fed with fish meat with and without MCs for 28 days [48]. In addition, in vitro investigation indicated that enzyme concentrations in blood including glutathione, superoxide dismutase (SOD), catalase (CAT), glutathione peroxidase (GPx) and glutathione s-transferase were altered. Increased hemolysis and pathological alterations in agglomerated and jagged erythrocytes were observed [53]. All these aforementioned results demonstrated that MC-LR possesses blood toxicity. 

Vascular endothelial cells are between blood and vascular wall tissue, which are composed of a layer of monocytes. Endothelial cells are the key regulators of vascular dynamic equilibrium and have an involvement in cell mitosis, vascular regeneration, vascular osmotic pressure, inflammatory response and platelet activity [117,118]. Therefore, any change in the structure and/or function of vascular endothelial cells may cause a destruction of the vascular system [119]. HUVECs were treated with MC-LR at a dose of 40 μM for 24 hours and the proliferation, migration ability and capillary-like structure formation of vascular endothelial cells were reported to be inhibited [50,51,52], whereas the apoptosis of endothelial cells, production of ROS, oxidative stress, expression levels of inflammatory factors and endothelial cell adhesion factors were found to be increased [50,51,52]. In another in vivo study, dysplastic, dysfunctional, destroyed structure, narrowed size of vascular and slower blood flow were found when zebrafish juveniles were co-cultured with MC-LR at a dose of 4.0 mM for 96 h [45]. The evidence thus suggests that MC-LR can induce vascular toxic effects. 

### 2.2. Indirect Cardiovascular Toxicity

The functions of mammalian organs usually intertwine. These organs are related to each other and affect and restrict each other. This implies that a dysfunction in one organ may cause a dysfunction in another organ or the abnormality of one organ may affect the normal function of another organ [54,55,56,57,58,59,60,61,62]. Similarly, MCs are capable of causing further CVD by inducing pathological changes in the structure and/or function of other target organs or tissues [21,22,23,24,25,26,27,31,32,33,54,55,56,57,58,59,60,61,62]—that is, the indirect cardiovascular toxicity of MCs. The following are studies demonstrating how abnormalities in other organs give rise to CVD.

#### 2.2.1. Liver Diseases Induced by MCs and CVD

The liver is one of the most vital target organs of MCs. MCs are transported into hepatocytes through OATP1B1 and OATP1B3, and exert hepatotoxicity [120]. A study carried out by Falconer et al. [121] showed that, under the induction of MC-LR, the cytoskeleton damage of isolated hepatocytes resulted in the loss of cell morphology, cell adhesion and cell death. The potential mechanism might be the inhibition of phosphatase, invoking hyperphosphorylation in a large number of hepatocytes, including keratin, which is responsible for microfilament orientation and intermediate filament integrity. As mentioned earlier, MC-LR can irreversibly inhibit the activities of PP1 and PP2A in hepatocytes, and may account for the disintegration of hepatocytes, apoptosis, necrosis, nonalcoholic steatohepatitis disease (NASH), intrahepatic hemorrhage, liver cancer and even animal death [122,123]. MC-LR may also induce hepatotoxicity by inhibiting the survival rate of hepatocytes, increasing hepatocyte apoptosis and ROS levels [124,125] as well as the activities of SOD, CAT, GPx and glutathione reductase in hepatocytes [126,127]. Several studies have shown that liver diseases are closely related to CVD. In a recent population survey, Mellinger et al. [128] reported that NASH may contribute to CVD including hypertension and atherosclerosis. Clinical data exhibited that NASH patients had higher carotid intima media thickness [129,130] and often experienced altered cardiac structure [131,132,133]. In addition, Boddi et al. [134] found that patients with ST-elevation myocardial infarction more often than not experienced NASH. The data suggest that MC-LR may induce NASH which has the potential to conduce CVD.

Krag et al. investigated 24 patients with liver cirrhosis and reported an increase in cardiac volume load and end-diastolic volume, as well as a decrease in left ventricular wall motion, CO and ejection fraction. Interestingly, myocardial perfusion was preserved [54]. Glenn et al. also exhibited a significant decline in diastolic reflux velocity, prolonged diastolic time and increased passive tension by assessing the relationship between liver diseases and CVD in cirrhotic animals [135]. Thus, liver disease is able to disrupt the cardiovascular system to cause diastolic dysfunction leading to CVD development. In a serum survey, Henriksen et al. [55] examined 51 patients with liver cirrhosis and revealed higher pro-BNP (pro-brain natriuretic peptide) and BNP (brain natriuretic peptide) in serum than those in normal subjects. Pro-BNP and BNP were generally indicators of abnormal QT-interval, heart rate and plasma volume in cardiac function [55]. In a recent study, Schimmel et al. [136] found that BNP levels in serum were related to cardiac function and severity of HF in patients with chronic heart failure (CHF). BNP levels were increased when cardiac systolic or diastolic dysfunction occurred, respectively, and, when both existed at the same time, the increasing levels were more significant [136]. Dong et al. [137] have also demonstrated that BNP levels in serum of cirrhosis were associated with ventricular wall thickness, diastolic dysfunction, stress-induced systolic dysfunction, hyperdynamic circulation and cardiac structural changes. Additionally, inflammation of liver, oxidative stress level of hepatocytes and apoptosis and proliferation of hepatocytes are able to increase the risk of CVD [138,139]. The evidence thus suggests that liver diseases caused by MCs can alter cardiac contractility, cardiac diastolic function and electrocardiograms through a variety of mechanisms, including autonomic nerve regulation, inflammation and changes in membrane channels, in order to give rise to CVD.

#### 2.2.2. Intestinal Diseases Induced by MCs and CVD

The gastrointestinal tract is another target organ of MCs. Oral exposure to MCs is the principal route of exposure [10]. After ingestion, MCs are absorbed into the gastrointestinal tract, transported into gastrointestinal epithelial cells mainly through OATP3A1 and OATP4A1 [31], and finally exert their multi-organ and multi-tissue toxicity through blood circulation. Studies revealed that partially unabsorbed MCs accumulated in the gastrointestinal tract can exert gastrointestinal toxic effects [32,33]. The earliest investigation on the intestinal toxicity of MC-LR was done by Falconer et al. [140] in 1992, by treating intestinal cells isolated from chickens with MCs. The intestinal cells exhibited time-and dose-dependent deformation or even death, and one or more blisters grew on the surface of deformed cells after the induction of MCs. The study also pointed out that the production of gastroenteritis related to ingestion of MCs may reflect the injury of intestinal epithelial cells caused by MCs. Botha et al. also in an in vitro study displayed that MC-LR could decrease the viability and induce time-dependent apoptosis of CaCo2 cells (an intestinal cell line) after the cell line was exposed to 50 μM/L MC-LR [141]. Furthermore, an acute in vivo study indicated that MC-LR could induce time-dependent apoptosis of duodenal, jejunum and ileum cells [78]. In view of this, MCs may have the ability to induce time-and dose-dependent toxic effects on the gastrointestinal tract.

Results of immunohistochemistry in vivo studies exhibited that MC-LR was mainly accumulated in intestinal microvilli [78], mucous layer, villi epithelial, lamina propria cells, and mucus secreted by the goblet cells of the small intestine [32], and in the cytoplasm and around the nucleus [142]. A comparative research explored by Gaudin et al. [143] adopted the method of feeding and I.P. injection of MC-LR for mice simultaneously. Comet assay suggested that both routes of exposure could remarkably increase the damage of intestinal DNA in mice, whereas the toxicity of I.P. injection of MC-LR was more serious. In summary, current explorations have shown that MCs induce gastrointestinal toxicity mainly by destroying the physical structure [143], the immune system [144], the balance of water and electrolytes in cells [143] and altering the activity of digestive enzymes in the chorion of the intestine [145], or inducing oxidative stress and apoptosis in intestinal cells [146,147,148] or even altering the intestinal microbes [149,150]. Meanwhile, there is considerable evidence that patients with inflammatory intestinal diseases have a higher risk of CVD [56,57]. In the early 1990s, Levine et al. [151] reported that HF belonged to a kind of chronic inflammation, and the expression levels of pro-inflammatory factors in serum were closely concerned with the morbidity rate of HF. Anker et al. [152] also indicated that a large number of inflammatory factors first originated from the intestinal tract. This suggested that the destruction of intestinal physical structure and immune system might transform the permeability of intestinal epithelial cells, and be conducive to uncontrolled entry and exit of substances into and out of cells, and finally flow into the various organs through blood circulation, leading to CVD. Data in recent years showed that animals displaying intestinal microbial alterations, bacterial translocation and the presence of bacteria in circulation after the destruction of intestinal barrier function brought about CVD such as vasculitis, hypertension, atherosclerosis and CHF [153,154,155]. 

#### 2.2.3. Kidney Diseases Induced by MCs and CVD

Although MCs are mainly accumulated in the liver and discharged through the bile duct, a small part of these toxins (about 9%) is filtered in the kidney and discharged through urine [156], which makes the kidney a potential target for MC toxicity. MCs can enter the kidney through OATP1 [157]. Menezes et al. [158] and Jia et al. [159] proved that MC-LR was capable of accumulating in the kidney and manifested its nephrotoxic effects. In a recent in vivo study, Wang et al. [160] confirmed the nephrotoxic toxicity induced by MC-LR. Zebrafish were treated with different doses of MC-LR and the pathological alterations in kidney tissue showed the existence of eosinophilic casts in renal tubules, abnormal renal tubules, decreased space intertubular and blood infiltration in renal cells. RNA-Seq analysis indicated disrupted renal gene expressions that had some involvement in various pathways, such as oxidative phosphorylation, cell cycle and protein processing in ER, concerned with apoptosis. TUNEL assay found the presence of renal cell apoptosis. Additionally, negative changes in the ROS level, apoptotic-related gene, protein expressions and enzyme activities revealed that MC-LR could induce production of ROS, subsequently triggering apoptosis via p53-bcl-2 and caspase-dependent pathway in the kidney. The data signifies that apoptosis may be a primary case of MC-LR-induced nephrotoxicity. Similarly, Nicole et al. in a recent review of the toxicology mechanism of MCs claimed that chronic exposure to low doses of MCs may pose a great risk to nephrotoxicity in mammals. MC-LR induced renal dysfunction, vascular and glomerular lesions and alterations in kidney tissues mainly by disrupting mitochondria and increasing ROS levels [161]. 

An in vitro exploration conducted by Dias et al. [162] demonstrated that MC-LR could induce the proliferation of renal cells through p38, JNK and Erk1/2 signaling pathways, leading to the emergence of renal tumors, after the monkey kidney cell line Vero-E6 were treated with different concentrations of MC-LR. Similarly, the human embryonic kidney 293 cell line (HEK293) and human kidney adenocarcinoma cell line (ACHN) were treated with different doses of MC-LR for 24 hours, and decreased viability as well as increased apoptosis of both cell lines were observed [27]. This suggests that MC-LR may contribute to chronic kidney disease (CKD) by damaging the renal cells.

Previous studies have shown that CVD is the most serious complication in patients with CKD, and its incidence rate is also increasing year by year. It is also the main cause of death in patients with CKD [58,59]. A cohort survey of patients with CKD indicated that CKD patients had higher blood pressure [60] and higher expression levels of biomarkers of inflammation-related factors, such as C-reactive protein, interleukin-6 and higher levels of endotoxin in serum, which can increase hemodynamic load and result in volume overload [61]. Moreover, due to long-term CKD, an iron metabolism disorder hinders the differentiation of red blood cells, and renal erythropoietin also decreases, leading to severe anemia in CKD patients [62]. A long-term increase in blood pressure, high expression levels of inflammatory factors, high volume load and anemia can inhibit the activation of xanthine oxidase, NADPH oxidase uncoupled nitric oxide synthase, and finally succeed in inducing oxidative stress as well as ROS level, decreasing antioxidant defense ability, nitric oxide and its activity, which give rise to left ventricular hypertrophy, vascular endothelial dysfunction, atherosclerosis and other CVD such as cardiac remodeling / fibrosis and HF [62,134].

## 3. Conclusions and Outlook

**Figure 3:**
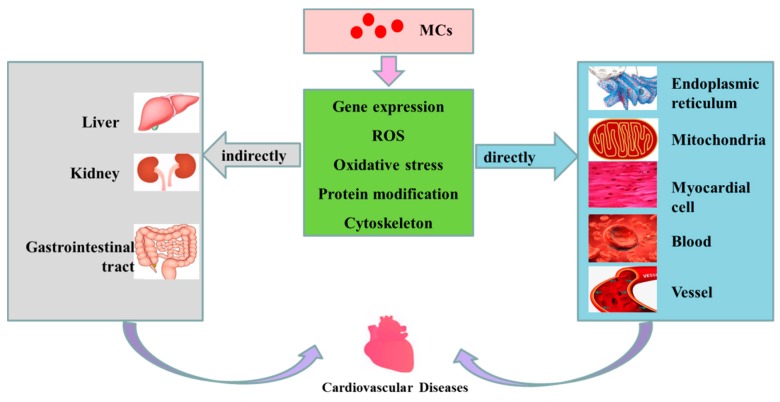
Possible mechanisms of cardiovascular toxicity caused by MCs. MCs are transported into cells through OATP (organic anion transporting polypeptides). Having entered the cells, MCs can cause alterations in expression level of some genes, increase the level of ROS, induce oxidative stress in mitochondria and ER, change the modification of related proteins (such as phosphorylation and dephosphorization of proteins) and destroy the cytoskeleton, subsequently playing direct or indirect toxicity to the cardiovascular system, and finally inducing the occurrence of CVD.

In this current paper, the effects of MCs on cardiovascular system were reviewed. Those studies have shown that cardiovascular toxicity is closely associated with MC exposure at various doses, pathways and in different species. Figure 3 outlines the possible mechanisms of cardiovascular toxicity of MCs. After exposure, MCs are transported into cells via OATP, and accumulated in the main organs and tissues. At this stage, MCs are preferentially concentrated in the liver, gastrointestinal tract, kidneys, cardiovascular and other organs. Having entered the cardiovascular system, MCs can induce cardiovascular toxicity directly by altering the morphology of cardiomyocytes, state of cellular apoptosis and proliferation, cytoskeleton, rhythm, the differential expression/activity of transcription factors, the ultrastructure, MMP and enzyme activity in respiratory chain in mitochondria of cardiomyocytes. MC exposure is also capable of raising the production of ROS and ER oxidative stress, resulting in cytoskeleton destruction, mitochondrial dysfunction and ER dysfunction. In addition, MCs are able to inhibit the activities of PP1 and PP2A, which lead to hyperphosphorylation of regulatory proteins, thus regulating cytoskeleton tissue, cell proliferation, apoptosis and CVD. MC exposure might also be associated with the damages of myocardial intercellular connection and vascular endothelial cellular cytoskeleton, or the reducing of cell vitality and increasing of apoptosis, oxidative stress levels as well as ROS levels in mitochondria and ER of blood cells and vascular endothelial cells. In addition to cardiovascular direct effects, MCs can indirectly induce CVD by destroying the structure and/or function of other organs including the liver, gastrointestinal area and kidneys. The review showed that exposure to MCs has influential toxic effects on the cardiovascular system of animals. Although there is no human data on cardiovascular toxicity of MCs, it is believed that cardiovascular toxicity caused by MCs may pose a great threat to human health, especially in view of the wide distribution and spread of MCs in the environment. 

It is of interest that the gap in understanding cardiovascular toxicity of MCs needs to be addressed through further research. 1. Direct data of exposure to MCs and the identification of MCs in human serum are necessary for human epidemiological studies of cardiovascular effects so as to understand the toxicology mechanism. 2. Whether maternal exposure to MCs during pregnancy affects the development of the heart of the offspring needs further exploration. 3. Do other congeners of MCs also have the potential to induce cardiovascular diseases in addition to MC-LR, MC-YR and MC-RR? 4. Other environmental factors including heavy metals, trace elements and organic pollutants could intensify or attenuate the toxicity of MCs. 5. Advanced and accessible technologies are essential to degrade and remove MCs from water. 6. States should be encouraged to establish acceptable maximum concentrations of MCs in drinking water, recreational water and irrigation water, especially in remote areas where rivers, lakes and streams are their main sources of water. Public education on the toxic effects of MCs and their related diseases should be strengthened. 7. Effective drugs that inhibit the binding of MCs to PP should be invented and manufactured. 8. Due to the elusive mechanism of cardiovascular toxicity of MCs, it is necessary to explore its molecular toxicology mechanism to develop targeted drugs. In addition, the toxicity of MCs to cardiac development needs to be further studied in order to prevent the occurrence of congenital heart disease. 

## Figures and Tables

**Table 1:** Summary of cardiovascular toxicity of microcystins *in vivo* studies.

Animals	Exposure	Toxicant	Dose	Time Point	Toxic Effects	References
Rabbits	I.P	MCs (mainly containing MC-RR, MC-LR	12.5, 50 μg/kg	1, 3, 12, 24, 48 h	Damage of mitochondrial morphology, lipid peroxidation ↑, SDH ↑, Ca^2+^-Mg^2+^-ATPase activities of mitochondria ↓, disrupted ionic homeostasis, MMP ↓	[36]
SD rats	I.P	MC-LR	122 μg/kg	1, 7, 14 day	Myocardial cells damage including pyknosis, plasma dissolve and myofibrilla necrosis, LDH ↑, AST ↑, CK ↑	[37]
loach, Misguruns mizolepis Gunthe embryos	orally	MC-LR	1, 3, 10, 100, 1000 μg/L	7 days	Pericardial edema, tubular heart and bradycardia	[38]
Wistar rats	I.P	MC-LR	10 μg/kg	every two days for 8 months	Destruction cytoskeletons of cardiomyocytes, loss of cell crossstriations, myofibril volume fraction ↓, cardiomyocytes volume ↑,myofibrillar volume ↓, even fibrosis, infiltration of mononuclear in the interstitial tissue	[39]
Male Wistar rats	I.P	MC-YR	10 μg/kg	every two days for 8 months	Volume density of cardiac muscle tissue ↓, fibrous proliferation, lymphocyte infiltration, enlarged and often bizarre-shaped nuclei, myofibril volume fraction ↓	[40]
Male Wistar rats	I.P	MC-LR	0.16 LD_50_ (14 g/kg), 1 LD_50_ (87 g/kg)	24 h	Myocardial infarction in dead rats, CK ↑, troponin I ↑, GSH ↑, lipid peroxides ↑, antioxidant enzymes ↑, heart rate ↓, blood pressure ↓, loss of adhesion between cardiomyocytes, swelling or rupture of mitochondria, complex I/III ↓, electron flow along the mitochondrial respiratory chain ↓	[41]
Zebrafish	orally	MC-LR	0.1, 1 μM	24 h	Angiodysplasia, damaged vascular structures, lumen size ↓, blood flow ↓, vascular dysfunction	[42]
Trahira, hoplias malabaricus	orally	MC-LR	100 μg/kg	48 h	GPx ↑, GSH ↓	[43]
Japanese medaka (Oryzias latipes)	orally	MC-LR	600, 6300 μg/L	15 days	Heart rate ↓, bradycardia	[44]
zebrafish juvenile fish	orally	MC-LR	4.0 mM	96h	Heart rate ↓, apoptosis ↑, morphological deformity, stunted growth	[45]
Male Fischer 344 Rats	orally	MC-LR	100 μg/kg		Cardiac output and stroke volume ↓, an acute hypotension responsive to volume, expansion with whole blood; heart rate ↓, oxygen consumption ↓, carbon dioxide production ↓, metabolic rate ↓, progressive hypothermia, acid-base balance changes	[46]
KM (Kunming) male mice	I.P	MC-LR	0, 3.125, 6.25, 12.5, 25 μg/kg/day	7 days	ALP ↑, LDH ↑, AST ↑, ALT ↑, RBC↓, HGB↓, HCT ↓, MCV -, MCH -, MCHC -, WBC ↑, Mon ↑, Gran ↑	[47]
Male Wistar albino rats	orally	MCs (mainly containing MC-LR, MC-YR, MC-RR, MC-LF, MC-LW)	25,000 μg/kg of food	28 days	RBC ↓, MCV ↑, MCH ↓, MCHC ↑	[48]

**Table 2:** Summary of cardiovascular toxicity of microcystins *in vitro* studies.

Cells	Toxicant	Dose	Time Point	Toxic Effects	References
H_9_C_2_ (rat cardiomyocytes)	MC-LR	10 μM	0, 4, 8, 12, 16, 20, 24 h	Expression levels of rhythmic genes ↓, antioxidant genes ↑	[49]
Human umbilical vein endothelial cells (HUVECs)	MC-LR	0.1, 1 μM	24 h	Apoptosis ↑, caspase3/9 ↑, mitochondrial ROS ↑, MMP ↓, p53 ↑, PCNA ↓	[42]
HUVECs	MC-LR	40 μM	24 h	Proliferation ↓, apoptosis ↑, migration ↓, capillary-like structure formation ↓, ROS ↑, NF-κB ↑, TNF-α ↑, VCAM-1 ↑, ICAM-1 ↑	[50]
HUVECs	MC-LR	40 μM	24 h	Cell death ↑, cell viability ↓, migration ↓, tube formation ↓, ROS ↑, NF-κB ↑, TNF-α ↑, IL6 ↑, SOD ↓, GSH ↓, MDA ↑	[51]
HUVECs	MC-LR	40 μM	24 h	MMP ↓, decreased levels of cytochrome c ↓, caspase-3/-9 ↑, ROS ↑, apoptosis ↑, NRF 2/ HO 1 ↓	[52]
Crucian carp erythrocytes	MC-LR	0, 1, 10, 100, 1000 nM.	1, 3, 12, 24, 48 h	GSH ↑, SOD ↑, CAT ↑, GPx ↑, GST ↑, GLT ↓, lipid peroxidate ↑, hemolysis ↑, AchE ↓, Ca^2+^-Mg^2+^-ATPase ↓, Na^2+^-k^+^-ATPase ↓	[53]
